# Influence of metabolic state and body composition on the action of pharmacological treatment of migraine

**DOI:** 10.1186/s10194-024-01724-3

**Published:** 2024-02-13

**Authors:** Noor Bruijn, Romy van Lohuizen, Malgorzata Boron, Mira Fitzek, Francesca Gabriele, Giada Giuliani, Laura Melgarejo, Pavel Řehulka, Gabriele Sebastianelli, Paul Triller, Simone Vigneri, Behiye Özcan, Antoinette Maassen van den Brink, the European Headache Federation School of Advanced Studies (EHF‐SAS)

**Affiliations:** 1 Department of Internal Medicine, Division of Vascular Medicine and Pharmacology, Erasmus MC, Erasmus University Medical Center Rotterdam, PO Box 2040, 3000 CA Rotterdam, The Netherlands; 2 Department of Neurology, University Hospital, Wroclaw Medical University, Wroclaw, Poland; 3 Department of Neurology, Charité Universitätsmedizin Berlin, Berlin, Germany; 4 Department of Applied Clinical Sciences and Biotechnology, Neuroscience Section, University of L’Aquila, L’Aquila, Italy; 5 Department of Human Neurosciences, Sapienza University of Rome, Rome, Italy; 6 Neurology Department, Vall d’Hebron University Hospital, Barcelona, Spain; 7 St. Anne’s University Hospital, Faculty of Medicine Masaryk University Czech Republic, Brno, Czech Republic; 8 Department of Medico-Surgical Sciences and Biotechnologies, Sapienza University of Rome Polo Pontino ICOT, Latina, Italy; 9 Casa Di Cura Santa Maria Maddalena, Neurology and Neurophysiology Service, Occhiobello, Italy

**Keywords:** CGRP, Metabolic disorders, Diabetes mellitus, Migraine, Obesity, Lifestyle

## Abstract

Migraine is a disabling neurovascular disorder among people of all ages, with the highest prevalence in the fertile years, and in women. Migraine impacts the quality of life of affected individuals tremendously and, in addition, it is associated with highly prevalent metabolic diseases, such as obesity, diabetes mellitus and thyroid dysfunction. Also, the clinical response to drugs might be affected in patients with metabolic disease due to body composition and metabolic change. Therefore, the efficacy of antimigraine drugs could be altered in patients with both migraine and metabolic disease. However, knowledge of the pharmacology and the related clinical effects of antimigraine drugs in patients with metabolic disease are limited. Therefore, and given the clinical relevance, this article provides a comprehensive overview of the current research and hypotheses related to the influence of metabolic state and body composition on the action of antimigraine drugs. In addition, the influence of antimigraine drugs on metabolic functioning and, vice versa, the influence of metabolic diseases and its hormonal modulating medication on migraine activity is outlined. Future exploration on personalizing migraine treatment to individual characteristics is necessary to enhance therapeutic strategies, especially given its increasing significance in recent decades.

## Introduction

Migraine is a disabling neurovascular disease with a global prevalence of approximately 15%, with its prevalence being highest in females [1]. The recurrent headache attacks in migraine are characterized by severe pain and can be accompanied by photophobia, phonophobia, nausea and vomiting, following the IHS classification [2]. Migraine has a tremendous impact on the quality of life of individuals, as well as on economic and social aspects of society [3]. Moreover, migraine can be accompanied by different types of comorbidities, leading to additional burden [4].

Multiple studies have shown a relationship between migraine and several endocrine and metabolic disorders, indicating new pathophysiological mechanisms of migraine [5]. Pro-inflammatory molecules, such as calcitonin gene-related peptide (CGRP) and certain cytokines are upregulated in migraine, as well as in obesity [5]. Thereby, transmitters and peptides released from the hypothalamus, involved in regulation of eating behavior, have been postulated to play a role in migraine pathophysiology [5]. This also applies to adipocytokines, involved in the regulation of body weight through metabolism and appetite [5]. Some studies have shown a possible role of the insulin receptor in the pathophysiology of migraine [5]. Furthermore, genetic and immune mechanisms may explain a bidirectional association between migraine and thyroid dysfunction [6].

The prevalence of metabolic disorders is increasing dramatically worldwide and will continue to increase due to multiple factors, including social, economic, behavioral and genetic ones [7]. Consequent complications contribute to a large portion of comorbidity and mortality [3]. The rise of metabolic diseases, coupled with its associated comorbidities, suggests that clinicians will encounter these patients more often in clinical practice. Endocrine disorders, defined by an imbalance of certain hormones, cause extensive individual variations of drug actions [8]. Additionally, body composition and metabolic changes might influence the clinical response to antimigraine drugs [8, 9]. While sex hormones are obviously an important part of the endocrine system that affects migraine as described elsewhere [10]. In this review, we will focus on the metabolic effects on migraine. However, information regarding the impact of metabolic disorders on the pharmacokinetics and pharmacodynamics of migraine drugs remains limited.

Given the clinical relevance, this review provides a general overview of the influence of metabolic state and body composition on the action and efficacy of pharmacological migraine treatment. We will systematically address (I) the association between migraine and various metabolic diseases, (II) the impact of metabolic disease and their medical treatment on pharmacokinetics and pharmacodynamics and therefore (III) the efficacy of antimigraine treatment in individuals with metabolic disorders and its interaction with hormonal modulating medication. Moreover, we emphasize the importance of further experimental and scientific research to increase the understanding of the impact of metabolic state on pharmacokinetics and pharmacodynamics of drugs in order to optimize therapies and, consequently, to improve the severity and frequency rate of migraine attacks in patients with coinciding metabolic disease.

## Methods

We searched the following databases: Pubmed, Embase, MEDLINE, Web of Science Core Collection, Cochrane Central Register of Controlled Trials, and Google Scholar. The searches were performed between June, 2023 and October, 2023. The following main keywords were used: migraine, obesity, diabetes mellitus, thyroid dysfunction, and CGRP. Besides, reference lists within the included articles were identified to find articles that had not been previously acquired. No restriction in the search process was applied to be as complete as possible.

## Obesity

### Association between obesity and migraine

Obesity is an abnormal or excessive fat accumulation that affects health and quality of life of individuals, with an estimated prevalence of 13.0% in the adult population (15.0% for women and 11.0% for men) and is associated with several comorbidities (e.g. cardiovascular disorders) [11]. Obesity is defined by the World Health Organization as having a body mass index (BMI) of at least 30 (or at least 23 for people of Asian descent) [11, 12]. The association between obesity and frequency of migraine attacks has been confirmed by several studies with a more significant correlation when abdominal fat increase was more prominent [5, 11]. Several studies showed that overweight/obese migraine patients experience a higher frequency, severity, duration, and disability of their headache attacks, compared to those with a BMI < 25 [5]. It should be noted that findings regarding the association between migraine prevalence and BMI are inconsistent and may be confounded by the fact that long-term treatment of migraine is often associated with weight changes, which may represent an important side effect of headache therapy [5]. Furthermore, obesity may be responsible for affecting the response to antimigraine treatments due to alterations in pharmacokinetics and pharmacodynamics [5].

Several mechanisms have been postulated to explain the link between migraine and obesity, such as the increased release of several vascular mediators (e.g. calcitonin-gene related peptide (CGRP)), adipokines and pro-inflammatory biomarkers (e.g. interleukins, cytokines) in both obesity and migraine [5]. Moreover, unhealthy lifestyle habits have been considered potential triggers to hypothalamic impairment and peripheral pathological changes (e.g. autonomic dysfunction) either in obese patients and migraineurs [5]. Plasma or blood levels of IL-1β, IL-6, IL-8, TNF-α, and C-reactive protein (CRP) as well as CGRP and substance P have been found to be increased both in migraineurs and obese subjects [5, 13]. Likewise, hypothalamic dysfunction, parasympathetic-sympathetic imbalance, impaired release of neurotransmitters, such as noradrenaline or dopamine, and increased circulating leptin levels, have been addressed as a potential link between migraine and obesity [13].

### Effect of obesity on pharmacokinetics and pharmacodynamics

A significant increase in BMI can influence physiological processes with a great impact on key organs that play a role in the pharmacokinetics and pharmacodynamics of drugs [9].

#### Absorption

Gut wall permeability is increased and gastric emptying is accelerated in obesity. However, no significant difference is found in bioavailability following oral dosing [9]. Also, after subcutaneous administration, the drug absorption rate or bioavailability seems to be unaltered in obesity [9]. A pharmacokinetic analysis of propranolol, commonly used in migraine prophylaxis, in obese and normal subjects indicated that the difference was not statistically significant, despite the presence of a trend toward higher bioavailability in the obese group (35% vs. 27%) [14]. Enhanced gut permeability and possible decrease in gut CYP3A4 activity is seen in obesity [15]. Although evidence should be provided by focused studies, it could be speculated that bioavailability is only significantly increased in case of extreme obesity, which needs to be confirmed in future studies.

#### Distribution

The volume of distribution (Vd) mainly depends on drug properties, such as molecular size, lipid solubility, degree of ionization and the ability of the drug to cross biological membranes, but it can be also influenced by tissue blood flow and plasma protein binding [16]. The effects of obesity on Vd are difficult to predict. An increased distribution of lipophilic compounds might be expected in obese subjects: the affinity of each drug for the excess adipose tissue, however, is unique and lipophilicity alone does not necessarily predict change in Vd [16]. Obesity may lead to impaired cardiac function, reducing tissue perfusion, whereas it does not impact on serum protein concentration and drug binding to albumin [16].

#### Metabolism

Achieving steady state, following repeated administration of a drug, is poorly affected by its chemical properties. It is mainly influenced by the degree of liver and kidney perfusion and by the ability of these organs to extract the drug from the bloodstream [9]. Obesity can alter the liver blood flow and act on enzymes that are involved in phase I and phase II reactions [9]. Obesity appears to enhance the function of some within the activity of cytochrome P450 isozymes, while the function of others is reduced by obesity [17]. An increased CYP2E1 activity, reduced by weight loss, and a mild reduction of CYP3A4 function are reported in obese patients [17, 18]. Glucuronidation and sulphation reactions increase proportionately to the total body weight [19]. The clinical relevance of these reactions seems to be negligible, not changing the elimination half-life of drugs [19].

Obesity is associated with a low-grade chronic systemic inflammation [20]. A rise in BMI promotes plasma lipid peroxidation and decreases antioxidant defenses, leading to increased oxidative stress that plays an important role in the development of comorbidities [20]. This inflammatory state could influence also the pharmacokinetics of drugs. Monoamine oxidases (MAO), involved in amine oxidation in adipose tissue, seem to have greater activity in obese rats compared to lean controls [21]. However, these results are not fully confirmed in humans. An in-vitro study analyzed MAO and semicarbazide-sensitive amine oxidase (SSAO) expression during differentiation of human pre-adipocytes: a dramatic increase in their mRNA and protein levels was documented only in lipid-laden precursor cells [22]. On the contrary, a significant reduction in the MAO activity was observed in the subcutaneous adipose tissue of obese subjects [23].

#### Elimination

Phase II conjugation reactions are generally increased in obese patients, mainly in morbid obesity [24]. In this light, a facilitation in drug clearance is expected. Surprisingly, recent studies on morphine showed higher glucuronide concentrations in obese than in normal subjects, suggesting a decreased elimination [24]. This finding may be explained by an altered drug transporter function in obesity. It is known that patients with non-alcoholic steatohepatitis (NASH) have impaired activity in multidrug resistance protein (MPR) 2 and 3: these transporters are involved in the transport of bile acids, anionic drugs and other different metabolites (such as glucuronides) from hepatocytes to the blood (MRP3) or hepatocytes to the bile (MRP2) [25]. In obese subjects, in which NASH is frequently observed, a similar alteration may be present, influencing transporter expression and elimination of glucuronides [25].

Regarding renal drug clearance, obesity may variably affect renal function. Obesity is associated with an initial increase in the glomerular filtration and tubular secretion processes, which can lead at first to an increase in renal excretion [9]. Nevertheless, obesity is a risk factor for the development of chronic kidney disease and a reduction of the glomerular filtration rate is commonly seen [9]. In addition, some frequent comorbidities, such as sepsis or diabetes, can further worsen renal function in obese patients [9].

#### Pharmacodynamics

Contrary to the pharmacokinetics, few studies analyzed pharmacodynamic changes in obesity. However, it is reasonable to expect differences in drug efficacy and toxicity that require careful dose adjustment. These parameters could be also influenced by co-existing comorbidities and the use of sedative agents. Due to the frequent occurrence of obstructive sleep apnea in obese subjects, hypnotic drugs, such as benzodiazepines or opioid analgesics, could have a negative effect reducing effective breathing. Since these considerations are hypotheses, future studies on pharmacodynamics in obesity are needed.

### Effect of anti-obesity drugs on pharmacokinetics and pharmacodynamics

Obesity treatment is primarily focused on lifestyle changes, including a diet that reduces excessive energy intake and improves dietary quality [26]. However, pharmacological agents can be necessary to promote a more considerable weight loss [26]. Glucagonlike peptide-1 (GLP-1) analogues and the pancreatic lipase inhibitor, orlistat, are the most commonly used agents and they have no effect on CYP450 isoenzymes [26]. GLP-1 analogues are primarily metabolized by the enzyme dipeptidyl peptidase-4 (DPP-4) and are excreted by the kidneys. GLP-1 analogues stimulate satiety and suppresses appetite, whereas orlistat reduces intestinal fat absorption and secondarily stimulates the secretion of GLP-1 [26]. The bupropion-naltrexone combination is used to prevent the vicious cycle of addictive over-eating, acting on central nervous system reward pathways by activating pro-opiomelanocortin (POMC). Bupropion, which selectively inhibits noradrenaline and dopamine reuptake, is a strong inhibitor of CYP2D6 activity [27]: care should, therefore, be taken when bupropion is administered in patients taking drugs metabolized by this cytochrome. The low-dose combination of phentermine and topiramate is allowed in obesity treatment for up to 12 weeks in the USA [26]. Although it has robust weight loss potential, this drug is not approved in Europe, mainly because phentermine is an amphetamine analogue [26].

### Efficacy of migraine treatment in obesity and its interaction with obesity drugs

**Fig. 1:**
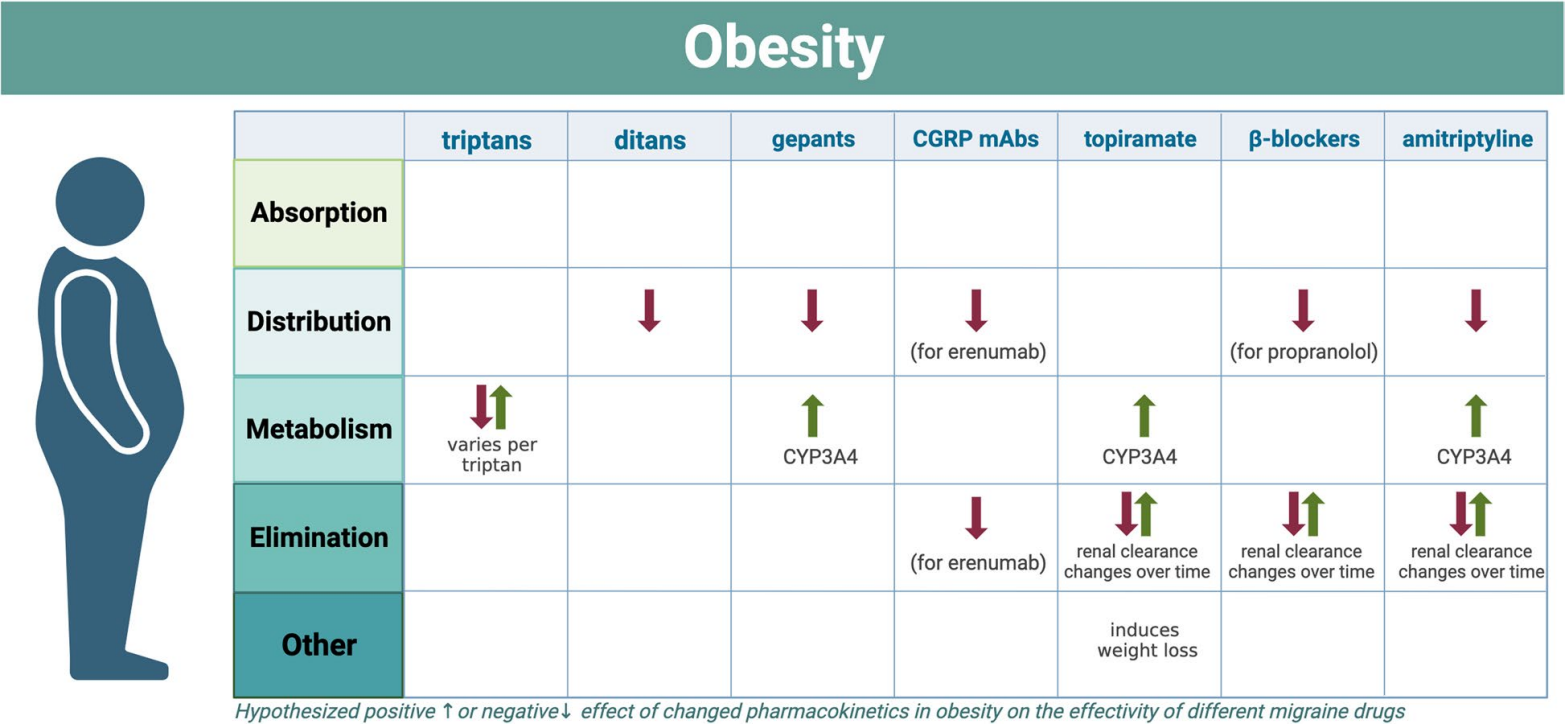
Overview of the influence of obesity on the efficacy of antimigraine drugs. *Absorption:* Gut permeability is increased and gastric emptying is accelerated in obesity, however more evidence regarding the effect of this on bioavailability is warranted. *Distribution:* Highly lipophilic drugs could be largely distributed in adipose tissue in obesity; Obesity may impair cardiac function, reducing tissue perfusion. *Metabolism:* CYP3A4 and CYP2C19 both show reduced activity in obesity. MAO activity might be increased, however more scientific research is needed to confirm this in humans; Obesity can alter liver blood flow. *Elimination:* Obesity is associated with an initial increase in renal excretion, followed by decreased renal excretion due to chronic kidney disease at later stages; Phase II conjugation can increase in obese morbid obesity

Comprehending the pharmacokinetic properties and potential drug interactions of antimigraine drugs is essential, since obesity and its medication have an impact on pharmacokinetics and pharmacodynamics. Based on the aforementioned information, the efficacy of migraine medications might be influenced by the presence of obesity and its medication (see Fig. 1). Thereby, known interaction profiles between the most common acute and prophylactic migraine medications and anti-obesity treatment are summarized.

#### Triptans

Triptans are selective and potent agonists of the vascular and neuronal serotonin (5-hydroxytryptamine, 5-HT) 5-HT_1B/1D(/1F)_ receptors [28–31]. 5-HT, a monoamine neurotransmitter, is involved in maintaining energy homeostasis, regulating behavior and suppressing appetite. Also, they induce vasoconstriction of meningeal vessels, reduce extravasation through dural vessels and inhibit the release of CGRP from presynaptic trigeminal sensory nerve endings [29–31]. Most triptans are quickly absorbed (T_max_ from 1 to 2.5 h) [32]. Subcutaneous administration of sumatriptan offers the highest bioavailability and quickest absorption [33]. All triptans are metabolized in the liver through various CYP-enzymes and/or MAO, which differ depending on the triptan [32, 34]. None of the triptans are highly lipophilic [35]. Triptans exhibit a greater than fivefold within-class difference in lipophilicity, with eletriptan being the most lipophilic, and sumatriptan the least [35]. Among the seven available triptans (almotriptan, eletriptan, frovatriptan, naratriptan, rizatriptan, sumatriptan, and zolmitriptan), eletriptan is the only one that is primarily eliminated through CYP3A4 isoenzyme (with a small contribution of CYP2D6). Also, frovatriptan and zolmitriptan are mainly metabolized by cytochrome P450 [35]. Other triptans are metabolized via other CYPs isoenzymes or predominantly via MAO [36]. Sumatriptan has a short half-life of approximately 2 h [37]. Except for rizatriptan, newer triptans degrade more slowly than sumatriptan, with frovatriptan having a plasma half-life of 26 to 30 h [38]. The elimination of triptans varies by subtype, but occurs primarily through a first-pass effect in the liver and via the kidneys [36].

We can expect that obesity could influence the serum concentration and thereby efficacy of the triptans due to changes in pharmacokinetics. Due to the possibility of increased MAO activity in obese persons, triptans could be metabolized more quickly in comparison with non-obese people. Also, lipophilic triptans could distribute largely in adipose tissue. In recent randomized controlled trials, the efficacy of different triptans in the acute treatment of migraine in non-obese versus obese patients was evaluated [39, 40]. The rate of pain free at 2 h was significantly larger in patients with normal BMI’s when compared to overweight subjects, regardless of the type of triptan [39]. However, lower rates of 48-h relapse were reported in both obese and non-obese patients, if treated with frovatriptan [39]. This effect could be explained, because obesity reduces the activity of cytochrome P450 and the extensive fat tissue serves as a depot. In another study testing the impact of weight and BMI in migraineurs treated with sumatriptan, higher systemic exposure to the medication was reported in subjects with lower values of BMI [40].

As mentioned above, 5-HT is involved in maintaining energy homeostasis [41]. In animal studies, the administration of 5-HT_1_ receptor agonists significantly reduced food intake [42]. Central serotonergic systems suppress feeding behaviors and depletion of central serotonin induces hyperphagia and body weight gain [42]. However, it must be noted that hydrophilic triptans are not likely to passage the brain-blood-barrier (BBB), whereas more lipophilic triptans (e.g. eletriptan) are more likely to passage the BBB, stimulating serotonergic systems [41]. To our knowledge, no direct interactions between triptans and GLP-1 analogues are known. In rare conditions, the use of bupropion and triptans may increase the risk of serotonin syndrome [43].

#### Ditans

Lasmiditan is a 5-HT_1_ receptor agonist with a more potent binding affinity for the 5-HT_1F_ receptor than the 5-HT_1B_ and the 5-HT_1D_ receptors and, thereby, inhibits neuropeptides, such as CGRP in the trigeminal ganglion [44]. It is highly lipophilic and, thus, capable of penetrating the BBB [44]. In contrast to the triptans, lasmiditan does not induce vasoconstriction, suggesting that the effect of 5-HT_1F_ receptor stimulation is mainly at the trigeminal nerve [44]. Upon oral administration, lasmiditan is rapidly absorbed with a T_max_ of 1.8 h [44]. The metabolism of lasmiditan is carried out by hepatic and extra-hepatic non-CYP enzymes [44]. Lasmiditan is eliminated via metabolism with a major pathway being ketone reduction. Renal excretion plays a minor role in drug clearance [44].

Considering the high lipophilicity of lasmiditan, an elevated BMI could contribute to lower its efficacy in migraine patients, due to distribution in fat tissue, which may serve as a depot. Secondly, lasmiditan is metabolized by non-CYP isoenzymes, which are not influenced by the presence of obesity [44, 45]. Thus, the metabolism of lasmiditan should not be influenced by the effect of obesity on pharmacokinetics. Importantly, the influence of body weight on the pharmacokinetics of lasmiditan is not fully understood yet and, thus, future studies are needed.

Studies testing weight changes following treatment with ditans are not yet available. To our knowledge, no direct interactions are known between ditans and GLP-1 analogues. The use of both bupropion and ditans could theoretically increase the risk of serotonin syndrome [43].

#### Gepants

Gepants (ubrogepant, atogepant, rimegepant, zavegepant) are small molecules that antagonize CGRP receptors [46]. The beneficial effect of gepants in a migraine attack might include the inhibition of the CGRP-induced vasodilation without causing vasoconstriction, and may also depend on neuronal effects [46]. Gepants are taken orally, expect for zavegepant, which is administered intranasally [46]. Gepants are quickly absorbed (T_max_ from 1–2 h) and, their C_max_ is approximately 1.5 h [46]. Plasma protein binding varies from 86% till 98% [46]. Due to their high lipophilicity, they are able to cross the BBB. They undergo extensively hepatic metabolism primarily by inactivation through CYP3A4 isoenzymes (for rimegepant, in addition, a lesser extent is metabolized by CYP2C9) [44, 47]. The elimination of gepants occurs fundamentally through faeces with minor renal elimination [44].

Gepants are highly lipophilic and, therefore, obesity could influence their pharmacokinetics. We expect that lipophilic gepants distribute largely to adipose tissues, resulting initially in lower plasma concentrations because of a larger distribution volume, and later perhaps in accumulation in adipose tissue. On the other hand, such accumulation could be beneficial, as it would provide a long-term depot [47]. As mentioned above, gepants undergo metabolism primarily by CYP3A4 enzymes. Since obesity reduces the activity of CYP3A4, we could expect higher serum concentration in patients with obesity. We can also expect altered hepatic blood flow in obesity, which may impact the elimination phase of gepants. However, several studies showed that body weight did not have any clinically relevant impact on systemic exposure (AUC and C_max_) to ubrogepant and rimegepant, which may possibly be explained by opposing effects of obesity on distribution volume and metabolizing enzymes [44], as reported in the US FDA report [47].

Studies testing weight changes following treatment with gepants are not yet available. To our knowledge, no direct interactions are known between gepants and GLP-1 analogues or bupropion.

#### CGRP (receptor) monoclonal antibodies

CGRP (receptor) monoclonal antibodies (mAbs) are one of the few specifically developed migraine prophylactic treatments [48]. They target the CGRP-molecule (fremanezumab, galcanezumab, and eptinezumab) or its receptor (erenumab) [48]. Their oral absorption is limited due to gastrointestinal degradation and inefficient epithelial diffusion [48]. Following subcutaneous (fremanezumab, galcanezumab, erenumab) or intravenous (eptinezumab) administration, antibodies are systemically absorbed through convective transport via lymphatic vessels [48]. The maximum serum concentration is achieved around 5-7d post-administration [48]. They are metabolized by general proteolytic degradation pathways. CGRP (receptor) mAbs are eliminated through intracellular catabolism primarily by the reticuloendothelial system [49]. Since mAbs do not undergo glomerular filtration due to their size, the effect of renal or hepatic dysfunction on plasma half-life is negligible [50].

In a post hoc pharmacokinetic analysis on seven clinical trials with galcanezumab, bodyweight, but not sex and race and ethnicity, modestly affected the pharmacokinetics of galcanezumab [47]. Also, increased body weight was associated with increased erenumab clearance and Vd [51]. As these effects were small, no dosage adjustments were recommended yet [51]. Obesity emerged as a negative predictor of CGRP (receptor) mAbs responsiveness in patients with chronic migraine [52]. This might be due to an increased CGRP activity characterizing obese migraineurs that mAbs would not be able to properly counteract [52].

No effect on body weight has been reported with CGRP (receptor) mAbs in migraineurs, although mAbs have recently been addressed as candidates for obesity treatment in clinical trials [53]. Lastly, interactions of the mAbs with other drugs are limited [54].

#### Topiramate

Topiramate acts through various pharmacological mechanisms, including modulation of electrolytes, enhancement of GABA activity, and inhibition of AMPA and kainate-mediated conductance [55–57]. It is highly bioavailable (around 80%) after oral administration, reaching peak plasma levels within 2.5 h and is distributed mainly in body water with a plasma protein binding of only 13–17% [58]. Topiramate is minimally metabolized by hydroxylation, hydrolysis and glucuronidation and acts as an inhibitor of cytochrome P450, CYP2C19, and as a mild inducer of CYP3A4 [59]. This could potentially cause pharmacological interactions [59]. It is excreted by the kidneys with an elimination half-life of 19–25 h [58].

Topiramate undergoes metabolism primarily by CYP450 enzymes [59]. Since obesity reduces the activity of CYP3A4, we could expect higher serum concentrations in patients with obesity. Furthermore, we expect that obesity does not affect the distribution of topiramate, since it distributes mainly in body water. Obesity increases the risk of renal diseases, which in turn can influence the elimination of topiramate since it is excreted by the kidneys.

Topiramate is the only migraine prophylactic medication linked with weight loss and has safely been used in experimental animal models as well as in clinical setting in the treatment of food disorders such as binge eating syndrome associated with obesity [60]. However, it must be noted that the European Medicines Agency’s committee recommends avoidance topiramate during pregnancy and breast feeding due to increased risk of neurodevelopmental problems after exposure [61]. Modulation of gamma-aminobutyric acid (GABA) receptors, causing glutamate inhibition as well as increased dopamine release have been postulated as main factors responsible for weight loss in patients treated with topiramate [62]. Moreover, a significant direct correlation has been found between circulating leptin levels and weight changes during topiramate treatment in subjects with partial onset seizures: nevertheless it is not clear whether the reduction of serum leptin may be the cause or the consequence of body fat mass loss [63].

#### β-blockers

Β-blockers inhibit the effects of (nor)adrenaline on β-receptors with a competitive antagonistic action [64]. Most β-blockers are absorbed through the gastrointestinal tract, reaching peak plasma concentrations in 1–3 h [65]. Lipophilic β-blockers are highly bound to plasma albumin, have short plasma half-lives, with a low bioavailability, being widely distributed to body tissue and penetrate the blood brain barrier, causing neurological side effects [65]. They undergo liver clearance, with hepatic “first-pass” metabolism, mediated primarily by the CYP2D6 enzymes [66, 67]. Hydrophilic β-blockers have a low plasma albumin binding, longer elimination half-lives, more stable plasma concentrations and do not cross the BBB, with less neurological side effects [64]. They are minimally metabolized by the liver, as they are eliminated unchanged by the kidney [64].

Propranolol, a β-blocker, is a lipophilic molecule, so its distribution could be influenced by obesity. Considering that propranolol is mainly metabolized by CYP2D6, its first-pass metabolism should not be influenced by obesity.

β-blockers seem to be more effective in decreasing blood pressure in obese than in lean hypertensive individuals, although the causes are still unclear and data on people with migraine are not available [68].

#### Tricyclic antidepressants

Amitriptyline, a tricyclic antidepressant (TCA), interacts with various receptors and acts as a sodium, calcium, and potassium channel blocker. Due to extensive first-pass metabolism, its bioavailability ranges from 20%-70% [69–71]. Amitriptyline has a high lipophilicity and therefore a high protein binding rate of approximately 95% [72]. The plasma half-life ranges from 10-28 h [72, 73]. Amitriptyline metabolism involves complex pathways, including demethylation mediated by CYP2C19 and hydroxylation mediated by CYP2D6, along with other isozymes like CYP3A4, CYP1A2, and CYP2C9 [74, 75]. Genetic polymorphisms can impact its metabolism, resulting in elevated plasma levels [76]. Amitriptyline is primarily eliminated by the kidneys [75].

We can expect that the prevalence of obesity could influence the serum concentration and efficacy of amitriptyline due to changes in distribution and metabolism. Amitriptyline is primarily metabolized to nortriptyline by CYP3A4 and CYP2C19, both of which have reduced activity in obesity [75]. Thus, concentration levels of amitriptyline could be influenced in obesity. Due to its high lipophilicity profile, amitriptyline could be largely distributed in adipose tissue.

Several studies on depressive disorders reported a relevant inverse correlation between patients’ high BMI or obesity and treatment response to various antidepressants [77]. The effects of TCA on weight are well documented, with more significant weight gain if amitriptyline or nortriptyline were administered [78]. Increased serum levels of leptin have been found following long-lasting use of amitriptyline and the calcium channel blocker, flunarizine (FZ) [79]. In another study, low plasma orexin-A and orexin-B levels and high neuropeptide-Y levels following the administration of amitriptyline and flunarizine were found [80]. Orexin-A levels were also negatively correlated to weight gain [80].

### Short summary

High BMI and obesity are associated with higher circulating levels of CGRP, substance P and leptin, but reduced concentrations of adiponectin, which in turn may be linked to a pro-inflammatory state subsequent to the excessive production of proteins such as TNF-α. A dysregulation in the hypothalamic activity has also been postulated as a possible link between obesity and migraine, due to the concomitant role of the hypothalamus in the regulation of body temperature, sleep, food and water intake, as well as in starting, maintaining, and ending of migraine attacks [5]. The significant impact of weight gain on boosting migraine severity seems in agreement with trials reporting improvement in frequency, disability or severity of the headache pain following surgical or dietary correction of weight [81]. Likewise, weight may have an impact on the efficacy of migraine prophylactic treatments due to changes in pharmacokinetics and pharmacodynamics with better responsiveness and more favorable outcome in patients with a lower BMI [82].

## Diabetes mellitus

### Association between diabetes mellitus and migraine

Migraine and diabetes mellitus (DM) are widespread worldwide, both representing a significant public health concern that affects people of all ages and genders [5]. Diabetes has been recognized as an epidemic due to its increasing incidence [5]. Some studies have shown that migraine patients exhibit impaired insulin sensitivity and elevated insulin levels, suggesting a possible role of the insulin receptor in migraine pathogenesis [83, 84]. This hypothesis is further supported by the association between migraine and polymorphisms in the insulin receptor gene [85]. Furthermore, CGRP, involved in migraine pathophysiology, has been found to be decreased in individuals with DM [86]. A population-based study reported a reduced prevalence of migraine among older diabetic patients, compared to the general population, suggesting that the seemingly protective effect of diabetes on migraine could be a result of neuropathy [87]. In addition, a nationwide cohort study showed that prior use of antidiabetic drugs was associated with a significantly decreased risk of medically treated migraine and that treatment of diabetes could have a protective role on migraine [88].

Most studies suggest a protective effect of DM against migraine, with a lower migraine prevalence observed among individuals with DM [88–90], while other studies have found a similar incidence rate without a significant association between the two conditions [91]. On the other hand, a unique case–control study indicated that individuals with migraine were more likely to have DM compared to those without migraine [92]. Also, a recent review suggested that brain insulin resistance, resulting in a chronic mismatch between the brain’s energy reserve and expenditure, may be an important mechanism in the development of metabolic abnormalities that drive migraine chronification [93]. A recently published systematic review provided a comprehensive overview of these studies and concluded that the degree of heterogeneity and the lack of statistical estimates in several reviewed studies prevented a meta-analysis of the data, leaving the relationship between migraine and DM unresolved [94].

It is worth noting that not all studies have differentiated between type 1 diabetes (T1D) and type 2 diabetes (T2D), and limited research has investigated the connection between T1D and migraine. However, data from several cohort studies suggest that T1D is associated with a decreased risk of migraine [88, 95].

### Effect of diabetes mellitus on pharmacokinetics and pharmacodynamics

DM significantly affects the metabolism of carbohydrates, lipids, and proteins, potentially causing alterations in the biochemical pathways associated with drug biotransformation. While the impact of DM on pharmacodynamics of drugs is still a subject of debate, its influence on pharmacokinetics is well established. Specifically, DM can affect drug pharmacokinetics by modifying processes related to absorption, distribution, metabolism, and elimination [8].

#### Absorption

Long-term complications of DM often involve macrovascular and microvascular changes that ultimately affect gastric emptying rate and intestinal transit time. Indeed, DM patients have delayed gastric emptying with the consequence of a significant prolongation of gastric and intestinal transit time [96, 97]. These changes have the potential to affect the rate and extent of absorption of orally-administered medications, resulting in a decrease in their serum concentration. Interestingly, Marangos et al. [98], did not demonstrate differences in oral bioavailability and the area under the concentration–time curve (AUC) between DM and non-DM patients.

For subcutaneous administration, differences exist between T1D and T2D. As example, the rate of absorption of subcutaneously administered insulin is strongly correlated with the subcutaneous blood flow. This, in turn, depends on the abdominal subcutaneous fat tissue [99]. Patients with T1D exhibit increased subcutaneous fat tissue blood flow [99], while T2D patients showed reduced subcutaneous fat tissue blood flow compared to healthy normal-weight individuals [100]. As a result, patients with T1D experience faster absorption of insulin, but no significant difference in drug bioavailability has been observed [101].

Microvascular changes in diabetics also affect muscle blood flow with a reduced rate of absorption for drugs administered intramuscularly [102].

#### Distribution

The Vd of a drug is closely linked to the amount of fat tissue present. Considering that obesity is a significant risk factor for the development of insulin resistance and DM, the Vd of lipophilic drugs in DM patients largely depends on the presence or absence of obesity [103]. Another important factor influencing distribution is albumin, which can be influenced by DM [104]. High glucose concentrations can cause a conformational change in albumin by the non-enzymatic glycation [104], potentially altering the free fraction of acidic drugs [105]. Moreover, drug binding to albumin can be reduced in a linear relationship with the levels of free fatty acids, which are generally elevated in in DM [105]. This results in a significant increase in the unbound fraction of albumin and several important drugs, such as antimigraine drugs, valproic acid and propranolol [106, 107].

#### Metabolism

In patients with DM there is a high incidence of abnormal hepatic function, including conditions such as non-alcoholic steatohepatitis, macrovesicular steatosis, liver fibrosis/cirrhosis, and focal fatty liver, which can impact drugs biotransformation [108]. Both non-alcoholic fatty liver disease (NAFLD) and DM have been associated with decreased expression and activity of hepatic CYP3A4 [108]. In addition, T2D patients have elevated levels of interleukin-6 (IL-6) and tumor necrosis factor-alpha (TNF-α) which have been associated with the downregulation of cytochrome P450 (CYP) isoforms CYP3As and CYP2C19 [109]. Most of the research on this topic has been conducted in animal models of DM, so caution should be exercised when extrapolating findings from animal studies to predict changes in humans [110]. Uncontrolled DM can have contrasting effects on enzymes involved in the two phases of biotransformation, with an overall increase in CYP enzymes but a decrease in phase II enzymes [111]. Differences in CYP enzymes exist between T1D and T2D; total CYP content in hepatic biopsies was found to be increased in T1D and decreased in T2D [111]. Most of the studies have focused on CYP2E1, CYP3A4, and CYP1A2. Controversial results have been reported regarding CYP2E1 [112, 113]. No differences between DM and non-DM patients have been observed for the activity of CYP1A2 and CYP3A5 [8, 114]. However, reduced activity and expression of CYP3A4 have been reported in T2D patients [115]. A recent study, comparing obese T2D patients with non-T2D obese patients and healthy controls, investigated the in vivo activities and protein expressions of CYP2C9, CYP1A2, CYP3A, CYP2C19 [116]. They found that patients with T2D had a significant downregulation effect solely on CYP2C19 activity, but not on the other CYPs [116].

In contrast to phase I biotransformation, the impact of DM on phase II biotransformation remains poorly understood due to limited available data and conflicting findings. Available evidence suggests that diabetes is associated with excessive production of reactive oxygen species and impaired antioxidant defense, leading to increased oxidative stress [117]. In line with this, most of the studies have focused on enzymes with antioxidant properties, such as the glutathione-S-transferase (GST) superfamily, which have been found to be affected by DM [118]. Additionally, a decrease in protein expression of the drug transporters ABCA1 and ABCG1 was described in leukocytes of T2D patients, with a strong correlation with the level of glycemia [119].

#### Elimination

Nephropathy is a common long-term complication of DM, affecting approximately 40% of patients [120]. Through micro- and macrovascular changes, DM leads to renal hyperfiltration and increases the glomerular filtration rate (GFR) [121]. The effect of GFR on the elimination of drugs in DM patients has been reported in several studies that looked at antibiotics in the pediatric population. While the difference in serum levels of amikacin was less noticeable, serum concentrations of kanamycin and bekanamycin were lower in pediatric DM compared to non-DM patients [122]. On the contrary, the half-life and GFR of penicillin G were significantly higher in children with DM than in healthy controls [123].

#### Pharmacodynamics

Despite the extensive body of research concerning the influence of DM on pharmacokinetics, there is limited data regarding its effects on pharmacodynamics. Previous studies have primarily focused on the influence of DM on the pharmacodynamics of cardiovascular medications. Two studies investigating the chronotropic response of isoprenaline have provided evidence of a significant reduction in isoprenaline-induced heart rate in diabetic patients [124]. Similar trends have also been observed for atropine and propranolol [125]. Although the underlying mechanisms are still under investigation, hypotheses involving changes in tissue adaptation to insulin levels and altered G-protein function have been proposed [126, 127]. Nonetheless, it remains unclear whether these effects primarily stem from diabetes-induced alterations in pharmacokinetics rather than pharmacodynamics.

### Effect of antidiabetic therapy on pharmacokinetics and pharmacodynamics

Multiple and optional antidiabetic drugs are available. Metformin**,** α-glucosidase inhibitors, delays carbohydrate absorption, which might influence the absorption of concomitantly administered drugs [128]. However, no drug-drug interactions have been reported until now [128]. Furthermore, metformin use is associated with anemia and vitamin B12 malabsorption, which may be due to a metformin-mediated effect on small bowel motility [129]. Metformin is also excreted by the kidneys which means that patients who take drugs known to reduce renal clearance might be at increased risk of metformin toxicity [130]. Additionally, other antidiabetic drugs such as glinides may competitively inhibit CYP3A4 conversion of other drugs that are also metabolized by this enzyme, with a consequent increase of side effects [131]. Newer agents are thiazolidinediones, such as pioglitazone, which improve glycemic control by altering the transcription of genes including carbohydrate and lipid metabolism [132].

Insulin analogues have been developed to improve the management of diabetes by mimicking the natural secretion of insulin, thereby reducing fluctuations in blood glucose levels [133]. They display distinct properties regarding their absorption, distribution, metabolism, elimination, and impact on blood glucose levels [133]. Primary sites of clearance are the liver and the kidneys [134]. Although no specific drug interactions or effect on general pharmacokinetics/-dynamics have been reported, there are certain agents that can influence the required insulin dosage based on their hyperglycemic or hypoglycemic activity [134]. Agents with hyperglycemic activity, such as corticosteroids, diuretics, estrogens, oral contraceptives and thyroid replacement therapy, may increase the dosage requirements of insulin [135]. On the other hand, medications known to increase the risk of hypoglycemia, including fluoxetine, sulfonamide antibiotics, monoamine oxidase inhibitors, angiotensin-converting enzyme inhibitors, β-blockers, and alcohol, have the potential to reduce insulin requirements [135, 136].

### Efficacy of migraine treatment in diabetes mellitus and its interaction with antidiabetic hormonal medication

**Fig. 2:**
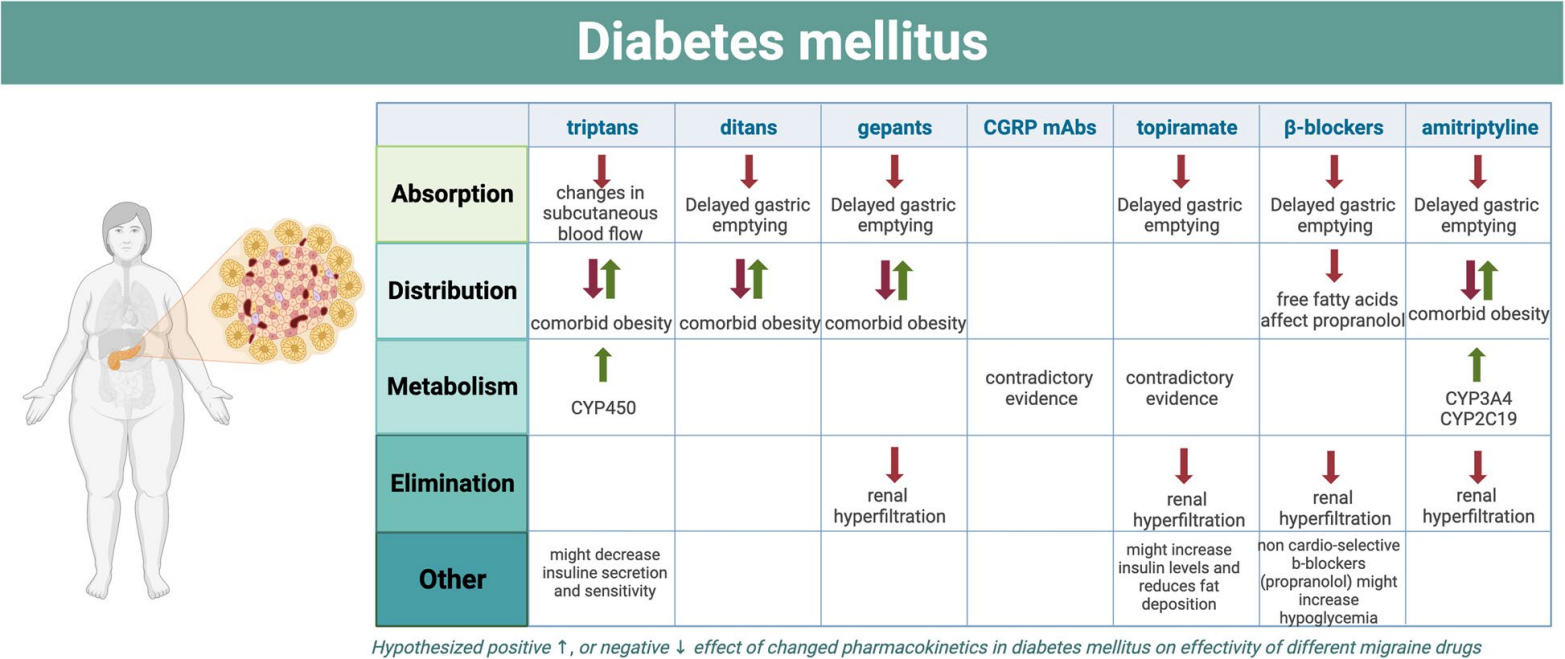
Overview of the influence of diabetes mellitus on the efficacy of antimigraine drugs. *Absorption:* Delayed gastric emptying in DM and changes in subcutaneous blood flow might cause decreased absorption. *Distribution:* Presence of comorbid obesity can increase the distribution of lipophilic drugs; Elevated free fatty acids in DM increases unbound fraction of propranolol. *Metabolism:* DM might downregulate CYP3A4 and CYP2C19, decreasing metabolism of some drugs; Concerning gepants and CGRP mAbs, contradictory evidence exists on the role of CGRP in glucose metabolism.* Elimination:* Renal hyperfiltration in diabetes mellitus can cause an increased elimination of renally eliminated drugs

Based on the aforementioned information, both types of DM and its medication have an impact on pharmacokinetics and pharmacodynamics. In patients with both DM and migraine, combined pharmacological therapy to treat migraine as well as optimize blood glucose levels is often required. This carries the risk of drug interactions, implying that the efficacy of migraine medications might be influenced by the presence of DM and its medication (see Fig. 2). However, there is a scarcity of research focused on the efficacy of antimigraine drugs in DM patients. Consequently, the real effects of DM on the efficacy of the different antimigraine drugs are largely speculative. Known interaction profiles between the most common acute and prophylactic migraine medications and antidiabetic treatment are summarized in the following paragraph.

#### Triptans

One of the most significant pharmacokinetic factors that could influence the efficacy of triptans (both for oral and subcutaneous routes of administration) is the lowering of the speed of absorption due to changes in macro-, and microvascularization induced by T2D [137]. Also, lipophilic triptans could widely be distributed in adipose tissue. Due to the possibility of decreased expression of cytochrome P450, triptans could be metabolized more slowly in comparison with those without DM.

In humans, sumatriptan may decrease plasma levels of somatostatin, glucagon, pancreatic polypeptide, insulin, and C-peptide [138]. It also decreases insulin levels after an oral glucose challenge without affecting glucose homeostasis [138]. In animal studies, the administration of 5-HT_1_ receptor agonists significantly reduced food intake, and plasma insulin levels were lowered without impairing glucose homeostasis [42]. In a study involving overweight in healthy women, a single 100 mg dose of sumatriptan resulted in reduced insulin secretion, insulin sensitivity, and glucose effectiveness [139]. Sumatriptan can influence pancreatic endocrine secretion, leading to changes in insulin levels and potentially impacting glucose metabolism [139].

The interaction between triptans and insulin is likely to be due to stimulation by triptans on serotonin levels and the resulting effect on insulin secretion. However, direct drug-drug interactions between triptans and insulin analogues have not been published yet. In animal models, there are no clinical reports in the big phase 3 trials or postmarketing suggesting a relevant risk of hypoglycemia or other interactions regarding blood sugar concentrations [139].

#### Lasmiditan

Lasmiditan is metabolized by non-CYPs isoenzymes or monoamine oxidase [44, 45], so its metabolism should not be influenced by effects of DM on pharmacokinetics. Apart from its direct central effects, lasmiditan inhibits CGRP release in peripheral and central trigeminal nerve terminals. Due to its lipophilicity, it crosses the BBB [140]. For more information with regards to antagonizing CGRP and its consequences on glucose management, we refer to section ‘*2.4.3 Gepants*’. Furthermore, no other direct drug-drug interactions between lasmiditan and insulin analogues have been reported.

#### Gepants

We expect that the pharmacokinetics effects of DM could influence the serum concentrations of gepants. For example, the delayed gastric emptying in DM could impact the absorption of gepants. Considering the high lipophilicity of gepants, an elevated BMI, as comorbidity in patients with T2D, is another factor that might contribute to lower their efficacy in migraine [47]. On the other hand, due to the impaired renal function in DM, higher serum concentration levels of gepants could be expected, complicating any predictions about serum concentrations of gepants in patients with DM.

CGRP is closely related to amylin, a key regulator in glucose metabolism that slows down gastric emptying, suppresses glucagon release and contributes to overall blood sugar control [141]. In a recent randomized controlled trial, amylin receptor agonism induced migraine attacks, supporting amylin as a contributor to migraine pathogenesis [142]. Although CGRP shares 46% amino acid sequence homology with amylin, the role of CGRP in glucose metabolism is somewhat contradictory [143]. αCGRP-deficient or antagonized mice displayed primary positive metabolic effects, such as increased β-oxidation and energy expenditure, as well as improved glucose control and insulin levels [144]. Further, reduced CGRP levels may improve glucose tolerance and insulin resistance [144]. Also, evidence from a human study stated that CGRP reduces insulin secretion [145]. These findings suggest that CGRP has a moderately negative effect on these metabolic parameters. Another study found that pharmacological dosing with CGRP resulted in conflicting data on glucagon and insulin levels as well as body weight in rodents [141].

Furthermore, research on direct drug-drug interactions between gepants and insulin analogues is lacking.

#### CGRP (receptor) monoclonal antibodies

The metabolism of mAbs against CGRP or its receptor is largely based on reticuloendothelial uptake while their degradation relies on enzymatic proteolysis into small peptides and amino acids [44]. Their large size prevents renal elimination and, after subcutaneous administration, they are absorbed and transported mainly through the lymphatic system [44]. Thus, renal and hepatic impairment are not expected to influence their pharmacokinetics [44, 47].

So far, there are no clinical trials focusing on the metabolic effects of CGRP (receptor) mAb treatments in humans. Due to overlapping agonism on CGRP receptors and amylin receptors, downstream effects are challenging to determine [44]. The coadministration of CGRP or CGRP receptor mAbs and concomitant drugs did not show clinically relevant pharmacokinetic interactions or negative effects on the drug-metabolizing enzymes or drug-transporters. This could be expected in view of the fact that the CGRP (receptor) mAbs are metabolized by general proteolytic degradation pathways [44].

#### Topiramate

We expect that DM could influence the pharmacokinetics of topiramate. Topiramate’s serum concentration could be elevated in DM patients due to impaired renal function and altered distribution. This could potentially lead to an increased likelihood of adverse events [146]. However, the delayed gastric emptying in DM might decrease the absorption of orally administered topiramate.

A number of studies have studied interactions between DM/DM medication and topiramate. Topiramate demonstrates a complex interaction with insulin, as observed in preclinical studies and human trials. In animal models, topiramate induces weight loss and acts as an insulin secretagogue and sensitizer in T2D rats, leading to improved glycemic control [146, 147]. In another animal study, topiramate increased insulin levels upon glucose tolerance testing, reduced fat deposition and the activity of lipoproteins in white adipose tissue, and demonstrated a direct protective action on β-cells against lipid-induced dysfunction [148]. Additionally, weight loss was consistently observed in most of the studies, with no dietary intervention [148]. While there was a moderate weight loss and improved glycated hemoglobin levels in T2D patients, a low dose of topiramate treatment had no significant effect on insulin sensitivity in obese females without established diabetes [149]. Topiramate seems to exhibit insulin secretagogue and sensitizing properties, however, the implications for its therapeutic use necessitate further investigation and consideration of potential severe side effects [150]. No evidence was found for a change in the pharmacokinetics of topiramate with concomitant insulin use. Previous research on potential interactions between topiramate and diabetes medication other than insulin reported a modest increase in metformin exposure and decrease in topiramate exposure following their coadministration. However, their concomitant intake is generally well tolerated [151]. The pharmacokinetics of topiramate at steady state are not affected by the intake of pioglitazone 30 mg [151].

#### β-blockers

Propranolol, a β-blocker, is a lipophilic molecule that binds with plasma proteins, including albumin and alpha-1 glycoproteins, for 80–95%. so its distribution could be influenced by DM [67]. In addition, as for the valproic acid, the circulation of free fatty acids increases the unbound fraction of propranolol, thereby influencing its serum concentration [106]. Considering that propranolol is mainly metabolized by CYP2D6, its first-pass metabolism should not be influenced by DM [67].

As for the interaction between β-blockers and antidiabetic treatment, the regulation of blood glucose depends on a complex relationship of hormonal and adrenergic influences [152]. Therefore, the use of β-blockers in patients taking antidiabetic drugs may result in potential adverse effects: prolongation of hypoglycemic episodes and bradycardia during hypoglycemic episodes, inhibition of some hypoglycemic symptoms and impairment of glucose tolerance with long-term use [152]. Especially non cardio-selective β-blockers are known to enhance hypoglycemia [153]. Although hypoglycemia is a well-known migraine trigger, it is worth noting that propranolol does not have a hypoglycemic effect in rat models [154]. Finally, patients concomitantly taking β-blockers and hypoglycemic drugs should be closely monitored to evaluate the effects on blood glucose levels [155]. On the other hand, meta-analysis of six large clinical trials showed that several β-blockers (bisoprolol, metoprolol, carvedilol) reduce the absolute mortality risk in diabetes patients [156].

#### Tricyclic antidepressants

Amitriptyline is primarily metabolized to the active metabolite nortriptyline by CYP2C19 [75], and in lesser extent by CYP3A4 and CYP2D6, which have reduced activity in DM patients [116]. Therefore, the efficacy of amitriptyline in migraine treatment may be influenced by DM, due to lower plasma concentrations of active metabolites [75].

Amitriptyline affects glucose and insulin metabolism through various mechanisms. It has been found to increase serum insulin levels, improve glucose tolerance and insulin sensitivity, and has found to show promising results by reducing glucose levels and improving insulin resistance in a mouse model of NASH [157]. A clinical study with patients being treated for depression also revealed increased insulin sensitivity and improvements in cholesterol metabolism parameters with amitriptyline use [158]. In a separate study, an amitriptyline-induced increase in blood glucose levels was reported, while insulin levels remained unchanged [159]. Although the effect of amitriptyline on glucose metabolism may imply a reduction in the required insulin dose, there is currently no evidence in the literature indicating direct drug interactions or mutual effects on the pharmacokinetics or pharmacodynamics between amitriptyline and insulin analogues or other antidiabetic drugs. In addition, our review of the literature revealed no evidence that insulin or other antidiabetic agents affect the pharmacologic properties of amitriptyline.

### Short summary

Among the preventive medication, the CGRP (receptor) mAbs are less affected by the pharmacokinetic effects of DM than the other, small molecular compounds. Amitriptyline, topiramate and possibly gepants have the advantage of improving glycemic control, but their metabolism is influenced by DM. However, amitriptyline leads to weight gain as a common side effect. Notably, mAbs could also improve glycemic control in patients with DM as this was observed in mouse studies. Considering that the speed of absorption significantly influences the efficacy of acute migraine treatment, the lowering of the speed of absorption induced by DM has the potential to reduce the efficacy of all acute antimigraine drugs. However, lasmiditan’s metabolism could be less affected by DM. While triptans (excluding eletriptan) are also a viable option, caution should be exercised regarding their use in individuals with cardiovascular comorbidities.

## Thyroid dysfunction

### Association between thyroid dysfunction and migraine

Thyroid dysfunction may be due to varying levels of thyroid hormones, such as thyrotropin-releasing hormone (TRH), thyroid-stimulating hormone (TSH), free triiodothyronine (fT3) and free thyroxine (fT4) [160]. Thyroid dysfunction is known to be one of the major endocrine disorders, with a prevalence 3%-21% worldwide [161]. Thyroid hormone plays a prominent role in metabolism and brain development in humans [162]. In the last decades, several epidemiological case–control studies confirmed an association between thyroid dysfunction and migraine, which is bidirectional [163]. Migraineurs are more prone to the development of new onset of hypothyroidism and, on the contrary, hypothyroidism may be associated with migraine, or increase severity and/or frequency of migraine attacks [164, 165]. Moreover, a significant genetic correlation with mutual single nucleotide polymorphisms (SNP) and gene loci has been found between migraine and thyroid dysfunction, particularly in hypothyroidism. This suggests that their complex association might be due to shared molecular genetic mechanisms [6]. Additionally, the treatment of subclinical hypothyroidism was effective in reducing both the frequency and severity of migraine attacks [166], which is also confirmed by a recent randomized controlled trial [167]. As opposed to hypothyroidism, little information about the association between migraine and hyperthyroidism is available.

Underlying mechanisms regarding the association between migraine and thyroid dysfunction remain unclear. A possible hypothesis is postulated for the role of the hypothalamus in migraine and thyroid function. The hypothalamus is in direct contact with vascular systems and functions both as a brain-endocrine organ as well as a connector to pain-modulating systems such as spinal trigeminal nuclei [168]. These nuclei play a major role in integrating and processing information to generate migraine attacks [168]. The pain transmitted from the spinal trigeminal nuclei to the hypothalamus may trigger neuro-endocrinological disbalances in migraineurs [169]. This leads to changes in the hypothalamically-regulated hormones, such as TSH, FSH and growth factors, which in turn, may contribute to the increase or decrease in thyroid hormone to cause thyroid dysfunction [169].

### Effect of thyroid dysfunction on pharmacokinetics and pharmacodynamics

As mentioned above, thyroid hormone significantly affects metabolism of carbohydrates, lipids and protein, and thus, potentially causing alterations in the biochemical pathways associated with drugs pharmacokinetics. Therefore, thyroid dysfunction may influence the response to treatments, such as for migraine, in absorption, distribution, metabolism and excretion.

#### Absorption

Disturbances in thyroid function affect gastrointestinal manifestations and gut motility. In hyperthyroidism, intestinal hypermotility reduces small bowel transit time, especially in patients with diarrhea [170]. Therefore, the rate absorption of substances or drugs is usually normal or may be decreased [170]. Interestingly, in spite of complete absorption of propranolol, it shows significant individual variation in bioavailability in patients with hyperthyroidism in comparison with euthyroidism [171]. This is thought to be caused by the extensive first-pass metabolism in the liver. Hypothyroidism induces a reduction of peristalsis and consequently constipation remains the most frequent gastrointestinal complaint [172].

#### Distribution

Controversial results exist in changes of Vd of drugs in patients with thyroid dysfunction. No significant changes in Vd were observed for digoxin and protein binding was unaffected in patients with hypo-, and hyperthyroidism [173]. An increased distribution of lipophilic compounds may be expected in patients with hypothyroidism due to hypometabolism and therefore as a consequence reduced energy expenditure and weight gain [174]. However, lipophilicity alone does not necessarily predict change in Vd. In hyperthyroidism, an increased cardiac output may change Vd positively, since it can be also influenced by tissue blood flow, whereas, vice versa, cardiac output may be decreased in hypothyroidism and, therefore, Vd may change negatively [175].

#### Metabolism

Thyroid hormones have shown to reduce the cytochrome P450 (CYP 3A4) activity in vitro. Liddle et al. [176], showed that T3 reduced CYP 3A4 enzymatic activity, messenger RNA, and protein expression in primary human hepatocytes. Also clinically, increased thyroid hormones reduce the activity of CYP3A4 [177]. This in turn, may influence the pharmacokinetics of concomitant drugs that are metabolized by the CYP3A4 enzyme. Further research is needed to evaluate the modification of the pharmacotherapy, dosage and drug selection in patients with elevated thyroid hormone levels. Also, other CYP enzymes may be affected by thyroid modulation. CYP7A1 is a rate-limiting enzyme in cholesterol metabolism and its expression is positively regulated by thyroid hormones [178]. Furthermore, levothyroxine may reduce MAO activity in rats [179, 180]. Elevated thyroid function was accompanied by decreased tissue levels of MAO [179].

On the other hand, animal data have shown that hypothyroidism can reduce the expression of various drug-metabolizing enzymes, such as CYPs, sulfotransferases and uridine 5-diphosphate-glucoronosyltransferases [181].

#### Elimination

Thyroid hormones play a substantial role in the regulation of body fluids, mainly affecting the renin-angiotensin system activity and renal haemodynamics (water and blood pressure homeostasis, natriuresis and other electrolytes economy) [175]. Renal clearance is elevated in hyperthyroid state due to increased cardiac output and renal blood flow, resulting in potentially decreased serum concentration levels of drugs. Renal clearance is decreased in hypothyroid state due to decreased cardiac output, resulting in potentially elevated serum concentration levels of drugs [175]. Thyroid hormones increase the already extensive presystemic clearance of propranolol and metoprolol [182].

#### Pharmacodynamics

Despite the extensive body of research concerning the influence of thyroid dysfunction on pharmacokinetics, there is limited data regarding its effects on pharmacodynamics. Thus, future studies are needed on the impact of thyroid dysfunction on pharmacodynamics.

### Effect of thyroid modulating hormonal therapy on pharmacokinetics and pharmacodynamics

When taken orally, levothyroxine is absorbed from the jejunum and upper ileum, with an absorption range of 40%-80% [183]. More than 99% of thyroid hormones are bound to plasma proteins, including thyroxine-binding globulin (TBG), thyroxine-binding prealbumin (TBPA), and albumin (TBA) [184]. FT4 has a higher affinity for TBG and TBPA, leading to slower metabolic clearance and a longer half-life compared to fT3 [184]. The metabolically active form is the free hormone. Sequential deiodination is the primary method of metabolism for thyroid hormones [185]. About 80% of fT3 is derived from peripheral fT4. The liver is the primary site for fT4 and fT3 degradation, although fT4 deiodination can occur in other sites, such as the kidney. fT4 is converted into equal amounts of fT3 and reverse T3 (rT3) through deiodination. fT3 and rT3 are further deiodinated into diiodothyronine [185]. Additionally, thyroid hormones can undergo conjugation and undergo enterohepatic recirculation. Thyroid hormones are primarily excreted by the kidneys. Approximately 20% of fT4 is excreted in the stool [185].

Thyroid hormones have significant effects on drug metabolism, and drug interactions can also impact thyroid function. fT3 has been shown to reduce the activity of CYP3A4 [177]. Hyperthyroidism can increase the clearance of certain drugs like antipyrin and β-blockers such as metoprolol and propranolol [182, 186]. Experimental hypothyroidism in rats leads to the induction of CYP3A2 and suppression of CYP2C11, while levothyroxine treatment decreases CYP8B1 activity. T3 can also affect the levels of other drug-metabolizing enzymes such as CYP2E1 and CYP1A [187]. Conversely, certain drugs like iodine-containing medications, lithium, and tyrosine kinase inhibitors can cause either hyperthyroidism or hypothyroidism [188, 189]. Additionally, drugs like phenobarbital, rifampin, and ritonavir can impact the metabolism or clearance of thyroid hormones [190–192].

### Efficacy of migraine treatment in thyroid function and its interaction with thyroid modulating hormonal medication

**Fig. 3:**
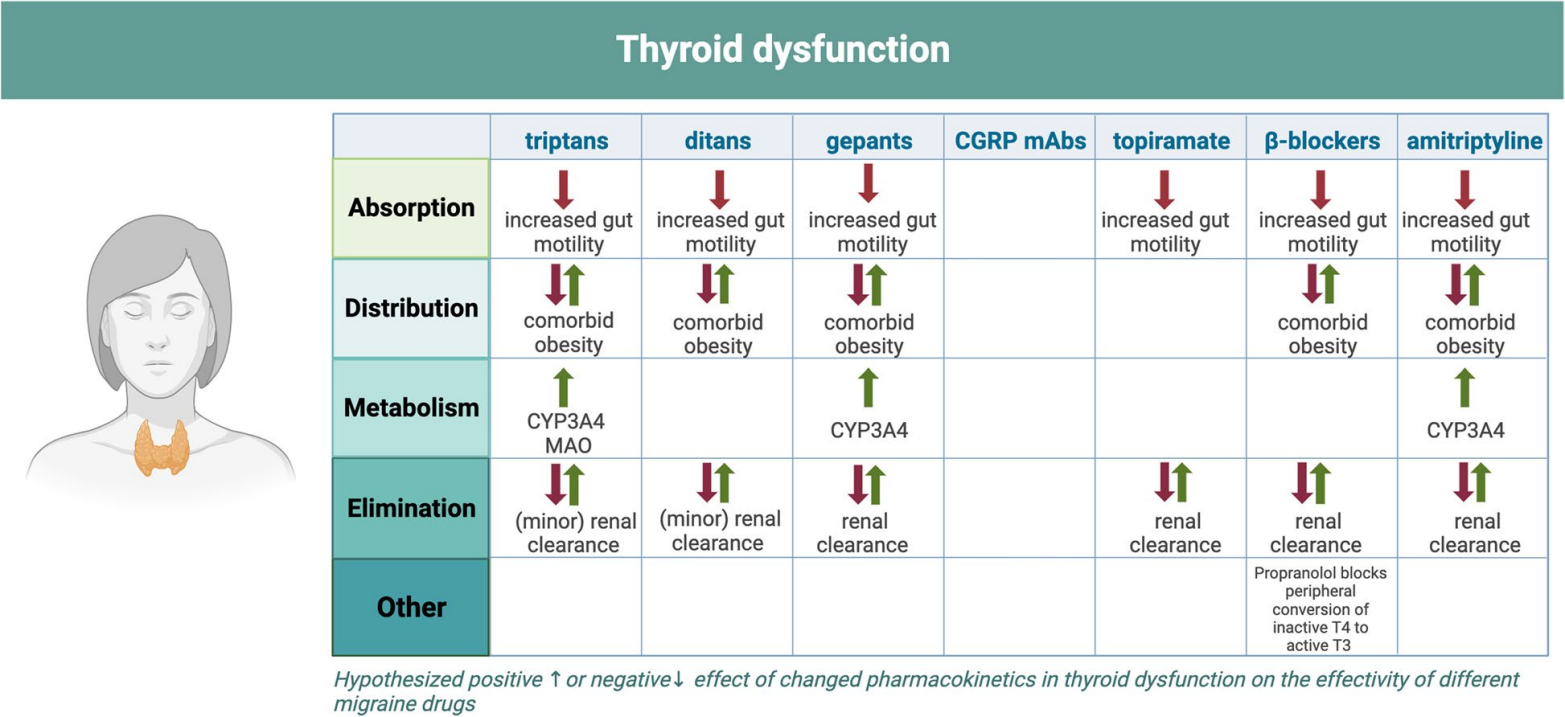
Overview of the influence of thyroid dysfunction on the efficacy of antimigraine drugs. *Absorption:* Increased gut motility in hyperthyroidism might cause decreased absorption. *Distribution:* Possible comorbid obesity in hypothyroidism may increase distribution of lipophilic drugs. *Metabolism:* Thyroid hormone reduces CYP3A4 and MAO, decreasing metabolism of some drugs; In hyperthyroidism propranolol is a preferred β-blocker, due to blockage of peripheral conversion of fT4 to fT3. *Elimination:* Renal clearance can be influenced by thyroid state via either an increased or decreased cardiac output in hyper- and hypothyroidism, respectively

Based on the aforementioned information, it may be assumed that thyroid dysfunction can affect migraine treatment (see Fig. 3). However, there is a scarcity of research focused on the efficacy of antimigraine drugs in patients with thyroid dysfunction. Consequently, the effects of thyroid dysfunction on the efficacy of the different antimigraine medications are still largely speculative. Additionally, known interaction profiles between the most common acute and prophylactic migraine medications treatment for thyroid dysfunction are summarized in the following paragraph.

#### Triptans

Due to the possibly reduced CYP3A4 enzymes and MAO in hyper- and hypothyroidism, serum concentration levels of triptans could be higher since they are metabolized by these enzymes. On the other hand, due to increased gut motility and risk of diarrhea in hyperthyroidism, the rate absorption of drugs may be decreased as well. In patients with hypothyroidism, the clearance of drugs may be decreased since the cardiac output, and therefore renal perfusion, may be decreased. Opposite effects may apply to patients with hyperthyroidism.

As for triptans and thyroid hormone supplements, no specific interactions or modulation of their pharmacokinetics and pharmacodynamics have been reported. Triptans are metabolized in part by cytochrome P450 (CYP) enzymes [35].

#### Ditans

Since lasmitidan is metabolized by non-CYP enzymes, its metabolism should not be influenced by thyroid dysfunction on pharmacokinetics. Since thyroid dysfunction may influence cardiac output and renal blood flow, we can expect changes in serum concentration of lasmitidan (*see *Sect. "Elimination"). No specific interactions between ditans and thyroid hormone supplements have been reported.

#### Gepants

Gepants are metabolized mainly by CYP3A-dependent pathway [46]. Thyroid hormones inhibit CYP3A enzymatic activity and, thus, we could expect higher serum concentrations in patients with hyper-, and hypothyroidism [177]. When taken orally, the rate of absorption may be influenced too. Gepants are eliminated through faeces. Since thyroid dysfunction may influence cardiac output and renal blood flow, we can also expect changes in renal excretion of these drugs (*see *Sect. "Elimination"). For gepants and thyroid hormone supplements, no specific interactions are reported.

#### CGRP (receptor) monoclonal antibodies

Since the metabolism of the CGRP (receptor) mAbs is largely based on reticuloendothelial uptake and the degradation relies on enzymatic proteolysis into small peptides and amino acids [44], we believe that thyroid dysfunction is unlikely to influence their pharmacokinetics.

The available literature does not report any specific interactions or alterations in the pharmacokinetics and pharmacodynamics for concurrently used CGRP (receptor) mAbs and thyroid hormone supplements. Considering a non-hepatic metabolism, potential cytochrome interactions modulated by thyroid hormones should not affect CGRP(R) mAb levels. Even though there are reports suggesting an increased phagocytic capacity in monocytes and macrophages mediated by high thyroid hormone levels, conclusive reports about the direct effect of thyroid hormones on mAbs are rare and need further investigation [193].

#### Topiramate

Alteration in serum concentration levels in patients with thyroid dysfunction might be expected, since topiramate is metabolized by CYP3A4 [59]. Moreover, hyperthyroidism can increase renal blood flow, and in turn, elevate the renal excretion of topiramate, and vice versa in patients with hypothyroidism (*see *Sect. "Elimination").

Findings on the interactions between topiramate and thyroid are contradictory. One study, by Shih et al. [194], in 2017 showed that topiramate resulted in reduced levels of T4 in epilepsy patients, with 30.6% exhibiting low T4 levels (< 0.89 ng/dL). However, a recent study indicated that topiramate did not induce any significant changes in thyroid hormone levels, including fT3, fT4 and TSH [195]. A recent meta-analysis suggested that topiramate can increase TSH levels while suppressing fT4 levels [196]. It is important to note that there was a limited number of studies with a small sample size. Besides that, there is no evidence for significant reciprocal effects of topiramate and thyroid hormones on the cytochrome P450 system regarding their bioavailability. Poor tolerability of topiramate can be expected in patients with hypothyroidism. A number of drugs are influencing CYP enzymes and reducing CYP3A activity can lead to aggravation of pre-existing migraine. Based on the information above we can assume that thyroid dysfunction, especially hypothyroidism, has an effect on antimigraine treatment with topiramate.

#### β-blockers

Thyroid dysfunction might well influence the pharmacokinetics of β-blockers, since they are absorbed through the gastrointestinal tract [64]. Thereby, β-blockers are eliminated by the kidney [64]. Since thyroid dysfunction may influence cardiac output and renal blood flow, we can expect changes in serum concentration (*see *Sect. "Elimination").

β-blockers are widely used both for migraine prevention, as for asymptomatic therapy of hyperthyroidism [197]. Propranolol is the preferred agent for β-adrenergic blockade due to its additional effect of blocking the peripheral conversion of fT4 to active form fT3 [197]. This happens via inhibition of the 5'-monodeiodinase that converts fT4 to fT3 compared to metoprolol [198]. Propranolol significantly improves the tachycardia, tremor, restlessness, anxiety, sweating, heat intolerance, and myopathy [64]. Propranolol decreases plasma fT3 and increases plasma rT3 in a dose-dependent manner [197]. These changes in circulating thyroid hormone levels are due to an inhibition of the conversion of fT4 into fT3 and of rT3 into 3,3’-T2 [199]. This effect of propranolol on the conversion of fT4 to fT3 has been demonstrated in vitro in studies of rat liver and kidney [200]. Furthermore, the decrease of plasma fT3 seems to determine some of the metabolic responses to β-blockers [197, 201].

#### Tricyclic antidepressants

Amitriptyline is metabolized by cytochrome P450 [75], so we may expect changes in serum concentration in patients with thyroid dysfunction. Also, amitriptyline is eliminated by the kidney [75]. Thereby, serum concentration levels may alter in thyroid dysfunction due to differences in cardiac output and renal blood flow (*see *Sect. "Elimination").

Both amitriptyline and thyroid hormones show a high protein binding rate [70, 184]. However, due to distinct binding partners, with amitriptyline predominantly binding to alpha-1-acid glycoprotein (AAG), and thyroid hormones binding to thyroxine-binding globulin (TBG), thyroxine-binding prealbumin (TBPA), and albumin, direct competition between the two drugs appears to be unlikely [184, 202]. Based on our literature search, no direct drug-drug interactions or mutual influences on the pharmacokinetics or -dynamics between amitriptyline and thyroid hormone supplements were found.

## Future implications

This review aims to provide a comprehensive overview of the worldwide impact of highly prevalent metabolic diseases (obesity, DM and thyroid dysfunction) on the efficacy of antimigraine drugs and vice versa. The intricate relationship between metabolic diseases and migraine is also discussed. Despite the extensive research regarding these topics, there is still a need for further research to understand these complex relationships in order to guide clinical practice effectively. Also, the potential impact of metabolic diseases on pharmacokinetics and pharmacodynamics of antimigraine drugs needs further exploration.

We encourage clinical physicians to monitor their patients with respect to a number of different factors that could affect pharmacokinetics of antimigraine drugs. Consideration of body weight, glucose impairment, and alterations in thyroid hormone are relevant parameters towards optimizing clinical antimigraine treatment. These variables could interact and could be relevant in patients with more resistance to antimigraine drugs. Obesity, DM and thyroid dysfunction can influence drug pharmacokinetics by modifying processes related to absorption, distribution, metabolism, and elimination. Mainly metabolism, a crucial component of drug processing, is influenced by reducing or increasing the activity of cytochrome P450 and MAO [18, 21, 109, 177]. However, the above should be considered as a hypothesis, given that few studies have been conducted with regards to this topic.

Until now, trials assessing the efficacy of various antimigraine drugs have mostly considered the impact of body weight. Specifically, some studies observed that patients with a normal BMI had significantly higher rates of pain relief after the use of triptans compared to overweight patients [39]. Also, bodyweight modestly affected the pharmacokinetics of CGRP (receptor) mAbs, such as galcanezumab and erenumab [47, 51]. Limited research on antimigraine drugs is available in patients with DM or thyroid dysfunction. In other words, the real effects of DM and thyroid dysfunction on the efficacy of antimigraine drugs with regards to pharmacokinetics are largely speculative and encouragement of future developments is needed to evaluate this. Next to this, the effects of metabolic disease on the pharmacodynamics with regards to antimigraine drugs are limited. Thus, future studies are needed to study this important topic.

Apart from the effect of metabolic state on antimigraine drugs, it has been suggested that preventive but also acute antimigraine drugs can in turn improve metabolic functioning [203]. Some antimigraine drugs are associated with weight loss, which may influence the activity of migraines, for instance topiramate, through modulation of GABA receptors [5]. Moreover, topiramate seems to exhibit insulin secretagogue and sensitizing properties, however, the implications for its therapeutic needs further investigation, considering its potential severe (congenital) side effects [61, 150]. In addition, triptans may influence insulin, glucose metabolism and regulation of appetite, resulting in weight loss and potentially impacting glucose metabolism [41, 42]. Data in humans regarding the effects of CGRP and gepants on the regulation of insulin, glucagon and body weight, is lacking and needs further research. While the use of β-blockers in patients taking antidiabetic drugs may result in potential adverse effects, an meta-analysis showed that β-blockers could reduce the absolute mortality risk in diabetes patients [156]. Thereby, β-blockers are also widely used for asymptomatic therapy of hyperthyroidism [197]. However, antimigraine drugs may also worsen metabolic disease. For example, while amitriptyline has the advantage of improving glycemic control, more significant weight gain is documented as common side effect [78].

Furthermore, optimizing and improving metabolic diseases through hormonal modulating therapies, may, in turn, also reduce migraine severity and frequency. It is important to emphasize that an improvement in lifestyle might reduce the frequency and severity of migraine attacks in obesity and DM, probably by affecting the central and peripheral mechanisms in migraine pathogenesis [5, 81]. The association between thyroid dysfunction and migraine is bidirectional, so hypothyroidism may cause migraine or increase severity and frequency of migraine attacks [164]. If hypothyroidism is treated and monitored correctly, the frequency and severity of migraine attacks may also decrease. This could also apply to the use of anti-obesity drugs and antidiabetic treatment [81, 88]. However, hormonal modulating therapies may also increase migraine activity. For instance, amylin receptor agonism induces migraine attacks, supporting the underlying pathological mechanism of amylin and CGRP in migraine [142].

Also, we addressed the potential interaction between antimigraine drugs and hormonal modulating therapy. Overall, no drug interactions were reported. Nevertheless, the use of both bupropion and triptans or ditans may increase the risk of serotonin syndrome [43]. Notably, β-blockers and antidiabetic drugs may result in hypoglycemic episodes and bradycardia [152]. Due to limited data, we emphasize the need for further research on the drug interactions between various antimigraine drugs, especially gepants and CGRP (receptor) mAbs, and hormonal modulating therapy.

## Conclusion

Migraine is a highly prevalent neurological disorder and many patients present with a broad variety of comorbidities [1]. Among the most common are widespread metabolic and endocrinological disorders such as obesity, insulin resistance or diabetes, and hypothyroidism. Therefore, an understanding of bioavailability, elimination pathways and potential interactions in pharmacokinetics via the CYP systems and MAO activity, is essential to establish optimal antimigraine treatment. The presence of alterations in CYP450 isoenzyme and MAO activity within patients with metabolic diseases should be evaluated, since several antimigraine drugs share metabolic routes by the CYP450 isoenzyme. This may result in potentially low efficacy of antimigraine drugs in case of CYP450 induction or, vice versa, in toxicity, in case of CYP450 reduction. Additionally, some preventive and acute antimigraine drugs can improve metabolic functioning. Vice versa, medication used to treat metabolic diseases may influence migraine activity and needs further exploration. Physicians should be aware of the influence of obesity on pharmacokinetics of antimigraine drugs. Notably, further research is needed to evaluate the potentially influenced efficacy of antimigraine drugs in patients with DM and thyroid dysfunction.

In conclusion, this review underscores the importance of the abovementioned interactions between metabolic state and disease, migraine, and different types of drugs used to treat these conditions. Further research into these complex interactions is warranted in order to optimize therapeutical strategies and, therefore, improve quality of life in migraine patients with metabolic disease.

## Data Availability

No datasets were generated or analysed during the current study.
